# 760 Building Cold Injury Prevention and Control Strategies with People Who are Experiencing Homelessness

**DOI:** 10.1093/jbcr/irad045.235

**Published:** 2023-05-15

**Authors:** Caitlin Orton, Mark Gannon, Barclay Stewart, Tam Pham, Aldina Mesic, Gretchen Carrougher

**Affiliations:** University of Washington, Seattle, Washington; University of Washington, Seattle, Washington; University of Washington, Seattle, Washington; University of Washington, Seattle, Washington; University of Washington, Seattle, Washington

## Abstract

**Introduction:**

The incidence of hypothermia and frostbite is increasing given larger numbers of people experiencing homelessness (PEH) across the country. Cold injuries create functional deficits that compound comorbidities of people who already experience disproportionate disability burdens. We collaborated with PEH to identify modifiable cold injury hazards, understand prevention knowledge and first aid techniques, and develop prevention strategies.

**Methods:**

We conducted 12 interviews with PEH with and without lived experience of cold injury. Interviews covered domains of risk factors/hazards, utility of existing educational materials, novel strategies, and acceptable stakeholders for dissemination. Interviews were transcribed and coded by two analysts using both deductive and inductive approaches.

**Results:**

Participants varied in the type (e.g., street, encampment, shelter) and chronicity of homelessness. Six themes emerged: i) PEH are engaged in prevention, ii) there is a need for improved awareness around synergistic risks (e.g., rain, nicotine or amphetamine use, diabetes), iii) existing materials are not sufficiently contextually specific, iv) a narrowly focused prevention and dissemination strategy will not suffice, v) prevention strategies need to be framed within the realities of living homeless, and vi) respect and kindness are integral to successful uptake (Table 1).

**Conclusions:**

Insightful perspectives shared by PEH provided key strategies for cold injury prevention. Prevention strategies must fit into the reality of homelessness, be respectfully delivered, and need to be a collective community effort where voices of PEH are valued.

**Applicability of Research to Practice:**

Themes and specific perspectives uncovered by this study can help clinicians, community service providers, and public health practitioners develop cold injury prevention strategies alongside people with lived experience of homelessness.

